# Two new species of the purse-web spider genus *Atypus* Latreille, 1804 from Hainan Island, China (Araneae, Atypidae)

**DOI:** 10.3897/zookeys.762.23282

**Published:** 2018-05-30

**Authors:** Fan Li, Xin Xu, Zengtao Zhang, Fengxiang Liu, Hongli Zhang, Daiqin Li

**Affiliations:** 1 Centre for Behavioural Ecology & Evolution (CBEE), College of Life Sciences, Hubei University, Wuhan, Hubei, China; 2 College of Life Sciences, Hunan Normal University, Changsha, Hunan, China; 3 College of Life Sciences, Hebei University, Baoding, Hebei, China; 4 Department of Biological Sciences, National University of Singapore, Singapore

**Keywords:** Atypidae, *Atypus*, DNA barcode, East Asia, Mygalomorphae, taxonomy

## Abstract

Two species of the purse-web spider genus *Atypus* Latreille, 1804 collected from Hainan Island, China, are diagnosed and described as new to science based on genital morphology, *A. baotingensis* **sp. n.** (♂♀) and *A. jianfengensis* **sp. n.** (♀). The DNA barcodes of the two species are also provided for future use.

## Introduction

**Figure 1.**
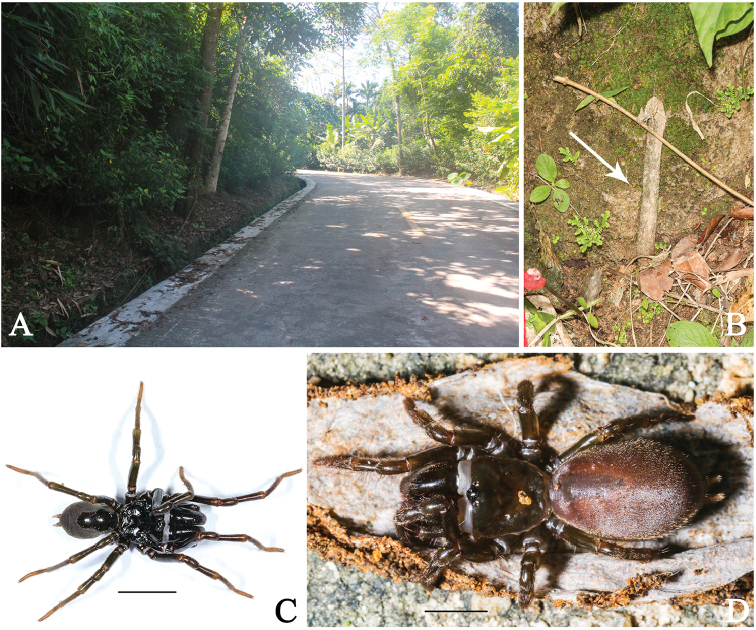
Microhabitat and general somatic morphology of *Atypus baotingensis* sp. n. **A–B** microhabitat **B** the purse-web, see white arrow point **C** male (HN-2017-037A) **D** female (HN-2017-032). Scale bars: 2 mm.

**Figure 2.**
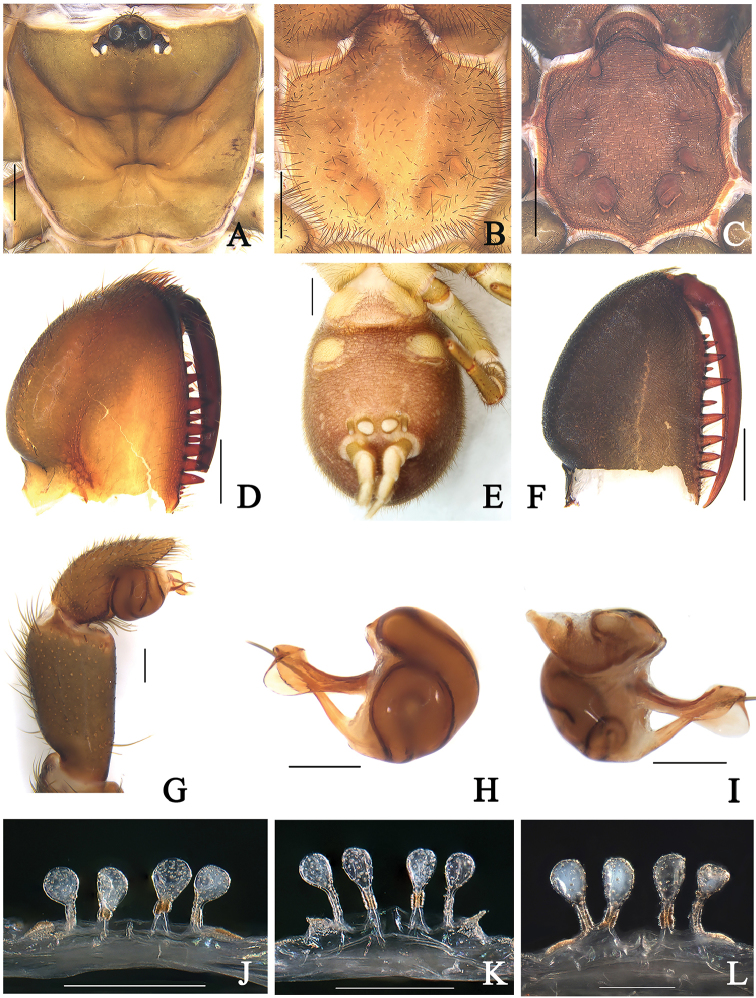
General somatic morphology and genital anatomy of *Atypus baotingensis* sp. n. **A–B, D–E, J** female (HN-2017-032) **C, F–I** male holotype (HN-2017-037A) **K** (HN-2017-033) **L** (HN-2017-036) **A** female carapace, dorsal view **B** female labium and sternum, ventral view **D** female left chelicera, inner-lateral view **E** epigyne and spinnerets, ventral view **J–L** vulva, dorsal view **C** labium and sternum, ventral view **F** left chelicera, inner-lateral view **G** left palpal, prolateral view **H** left palpal bulb, retrolateral view **I** same, prolateral view. Scale bars: 2 mm (**E**); 1 mm (**A–D, F**); 0.2 mm (**G–L**).

The purse-web spider family Atypidae is an ancient branch of the infraorder Mygalomorphae. Atypidae is one of the burrowing mygalomorph families. However, unlike other burrowing mygalomorph spiders, atypids form a tough web with silk from the end of their burrows to the upper ground section, which is expanded and camouflaged as a trap for wandering arthropods (Fig. 1) (Jocqué and Dippenaar-Schoeman 2006; Fourie et al. 2011). The family contains 52 species in three genera (*Atypus* Latreille, 1804, *Calommata* Lucus, 1837, and *Sphodros* Walckenaer, 1835) around the world. *Atypus* includes 32 species worldwide, of which, 13 are known from China (Zhang 1985; Schwendinger 1990; Zhu et al. 2006; Yin et al. 2012; Li and Lin 2016; World Spider Catalog 2018). *Atypus* can be distinguished from the other two genera as follows: male with marginal ridges in sternum; palp with short, straight, and spike-like embolus, as well as distally enlarged and straightforward conductor; female genitalia with bulbous or pyriform receptacula and with two lateral patches of pores on genital atrium (Fig. 2) (Schwendinger 1990; Zhu et al. 2006). Historically, Kraus and Baur (1974) gave detailed discussions on the taxonomic problems in *Atypus* species of Europe, and pointed out that the female genitalia are more reliable than the male bulb for atypid taxonomy. Schwendinger (1989) revised *Atypus* species in northern Thailand and also revised the whole genus in 1990 (Schwendinger 1990). Zhu et al. (2006) revised the genus *Atypus* of China. No new species has been described since the genus *Atypus* was revised a decade ago.

In this study, we diagnose and describe two new *Atypus* species collected from Hainan Island, China, using male and female genital morphology. To support our identifications in the future, here we provide COI barcode evidence. In addition, the female genitalia and male palp of the genus are illustrated for the first time with clear digital photographs.

## Materials and methods

All specimens were excavated from their underground silk tubes by the roadside (Fig. 1A). They were collected alive and fixed in absolute alcohol, their right four legs were removed for molecular work, and the remains were preserved in 75% ethanol for morphological work. Male palp and female genitalia were dissected using a stereomicroscope SZM 45-B2 (Ningbo Sunny Instruments Co., Ltd.). After being cleared with Proteinase K by being incubated at 56° C for 3 hours, female genitalia were photographed with the Olympus BX51 compound microscope using a MicroPublisher 3.3 RTV camera. The others were photographed with a Leica M205C digital microscope. All measurements were given in millimetres. All the specimens were examined and deposited in the Centre for Behavioural Ecology & Evolution (CBEE), College of Life sciences, Hubei University, Wuhan, China.

Abbreviations used:

**AL** abdomen length;

**ALE** anterior lateral eye;

**ALS** anterior lateral spinneret;

**AME** anterior median eye;

**AW** abdomen width;

**CL** carapace length;

**CW** carapace width;

**MOA** median ocular area;

**PLE** posterior lateral eye;

**PME** posterior median eye;

**TL** total length.

DNA barcodes were obtained for future use: a fragment of the mitochondrial gene cytochrome *c* oxidase subunit I (COI) was amplified and sequenced using the primer pairs: LCO1490 (5’-GGTCAACAAATCATAAAGATATTGG-3’) (Folmer et al. 1994) and HCO2198 (5’-TAAACTTCAGGGTGACCAAAAAATCA-3’) (Folmer et al. 1994). All molecular procedures on extraction, amplification and sequencing followed standard protocols (see Xu et al. 2015).

The genetic distance of the COI gene was calculated using MEGA version 6 (Tamura et al. 2013).

## Taxonomy

### Family Atypidae Thorell, 1870

#### Genus *Atypus* Latreille, 1804

##### Atypus baotingensis sp. n.

Taxon classificationAnimaliaAraneaeAtypidae

http://zoobank.org/B8795BAD-EF8F-4E4E-B032-908E91AFFBFA

Figs 1 , 2

###### Type material.

**Holotype male (HN-2017-037A)**: CHINA: Hainan Province: Baoting County, 2 km to Qixianling National Forest Park along y044 Road, 18.71°N, 109.68°E, 205 m elevation, collected on 21 August 2017 by X. Xu, F. Liu, Z. Zhang, and D. Li (CBEE).

###### Paratypes.

6 females (HN-2017-032-HN-2017-037) and 1 male (HN-2017-037B), collected at the same locality as the holotype, 21 August 2017 by X. Xu, F. Liu, Z. Zhang, and D. Li (CBEE).

###### Etymology.

The specific name refers to the type locality.

###### Diagnosis.

**Figure 3.**
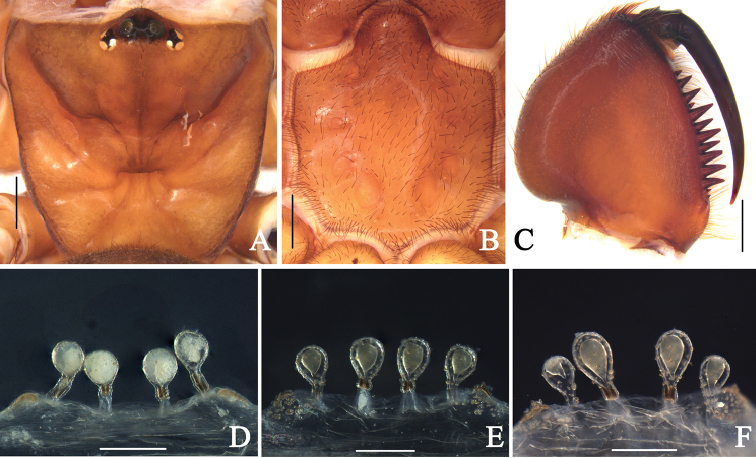
Genital anatomy of holotype and paratypes of *Atypus ledongensis*, see description details in Zhu et al. (2006). **A–D** female holotype (LD-001) **E–F** female paratypes **E** (LD-002) **F** (LD-003) **A** female carapace, dorsal view **B** female labium and sternum, ventral view **C** female left chelicera, inner-lateral view **D–F** vulva, dorsal view. Scale bars: 1 mm (**A–C**); 0.2 mm (**D–E**).

The male palp of this new species resembles that of *A. suiningensis* Zhang, 1985, but can be diagnosed from the latter by 1) the wide triangular space between its embolus and conductor in lateral views (Fig. 2H–I); 2) the relatively long flat upper margin of its conductor in retrolateral view, with a triangular folded part of the upper corner of its conductor (Fig. 2H); and 3) the first pair of sigilla are connected by arched wrinkles across the anterior sternal margin (Fig. 2C). The female genitalia of the new species resembles that of *A. ledongensis* (Fig. 3D–F), but can be distinguished from the latter by the basal stalks of median pair of receptacula almost being as long as those of the lateral pair (Fig. 2J–L). In *A. ledongensis*, the basal stalks of the median pair of receptacula are obviously short, whereas the basal stalks of lateral pair of receptacula are as long as their diameter (Fig. 3D–F).

###### Description.

**Male (holotype).** TL (including chelicerae) 11.44. CL 3.34, CW 3.55, AL 4.56, AW 2.78. Carapace black brown. Fovea placed back 2/3 of carapace length with some radiative grooves. Eye diameter: AME 0.24, ALE 0.10, PME 0.10. Distances: AME–AME 0.46, AME–ALE 0.31, PME–PME 0.83, PME–PLE 0.13. MOA 0.34, front width 0.94, back width 1.03. Labium wider than long. Sternum reddish brown, 3.11 long, 2.26 wide, moderately roughened clothed with fine black hairs. Sigilla deeply imprinted; first pair anteriorly pointed, close to the margin of the sternum; posterior pair oval bigger than other pairs; second pair small (Fig. 2C). Chelicerae black brown, with 13 teeth on promargin in a single row, basal three fairly small (Fig. 2F).

Abdomen grey black, oval, with dorsal scutum gloss black. Spinnerets six: ALS 0.41 long, PMS 0.72 long, four-segmented PLS with lengths as follows: basal 0.41, median 0.52, subapical 0.41, apical 0.33, total 1.67.

Palpal femur with furrow. Legs slender in red grey. Granular texture only on femur I present. Spines on all metatarsus; metatarsus IV with 17 dorsal spines. Leg formula: 1243.

Male palp (Fig. 2G–I): long conductor with a triangular folded part of its upper corner in retro-lateral view; embolus long, thin spike with a wide triangular space between embolus and conductor in lateral views.

**Female.** TL (including chelicerae) 15.91. CL 4.19, CW 4.02, AL 7.35, AW 5.34. Carapace black-brown. Eye region black. Eye diameters: AME 0.24, ALE 0.14, PME 0.18, PLE 0.17. Distances: AME–AME 0.29, AME–ALE 0.20, PME–PME 0.81, PME–PLE 0.09. MOA 0.48, front width 0.77, back width 1.17. Fovea transverse, occupying about 1/5 of carapace width at that point. Chelicerae orange brown with 13 teeth on the promargin in a single row, basal three fairly small. Sternum (Fig. 2B) light orange brown, 3.51 long, 2.30 width, smooth, with scattered black hairs; sigilla relatively light impressions, first pair anteriorly pointed; oval posterior pair much larger.

Abdomen, oval and medium brown (Fig. 1D), with indistinct oval dorsal scutum on anterior half. ALS 0.56, PMS 0.83, four-segmented PLS with lengths as follows: basal 0.66, median 0.70, subapical 0.59, apical 0.75, total 2.7.

Spines on all metatarsus; metatarsus IV with 13 dorsal spines. Leg formula: 4132.

Vulva (Fig. 2J–L): Genital atrium very short, pore patches small, receptacula attached to anterior edge of atrium; median pair with upper incrassate basal stalks and the basal stalks of the median pair almost being as long as the lateral pair; lateral pair attached to patches of pores.

**Table 1.** Leg measurements of *Atypus baotingensis* sp. n., male.

	Femur	Patella	Tibia	Metatarsus	Tarsus	Total
I	4.34	1.71	2.53	2.09	1.05	11.72
II	3.26	1.66	1.96	2.62	1.29	10.79
III	1.82	1.17	1.65	2.55	2.13	9.32
IV	1.83	1.39	2.39	2.66	1.70	9.97

**Table 2.** Leg measurements of *Atypus baotingensis* sp. n., female.

	Femur	Patella	Tibia	Metatarsus	Tarsus	Total
I	2.46	1.23	1.51	1.66	1.12	7.98
II	1.62	1.30	1.17	1.52	0.87	6.48
III	2.81	1.52	0.92	1.14	0.52	6.91
IV	2.71	1.61	1.36	1.89	1.21	8.78

###### Variation.

Size range of females: carapace length 4.19–5.12, carapace width 3.51–5.02, total length 14.13–16.91, n = 6; the basal stalks of left side pairs of receptacula connected in two specimens (Fig. 2L). Size range of males: carapace length 3.34–3.39, carapace width 3.23–3.55, total length 10.64–11.44, n = 2.

###### Habitat.

Purse webs were found attached to the soil slope along roadside (Fig. 1A–B).

###### Distribution.

Hainan Island (Baoting), China.

###### GenBank accession numbers.

HN-2017-032: MH279555; HN-2017-033: MH279556; HN-2017-036: MH279557; HN-2017-037: MH279558; HN-2017-037A: MH279559.

###### Remarks.

We examined the holotype and two paratypes of *A. ledongensis* (Museum of Hebei University, Baoding, Hebei, China), and also successfully sequenced the COI barcode of the holotype specimen (LD-001), which is available on GenBank (GenBank accession number MH279560). The lowest pairwise distance between the holotype of *A. ledongensis* and the specimens of *A. baotingensis* sp. n. is 7.2% in mean Kimura 2-parameter distance (K2P) and 6.8% in p-distance. The previous study revealed that the interspecific COI barcode for North American tarantulas is at 5% (Hamilton et al. 2014), thus it can guide us to diagnose our specimens as a new species, *A. baotingensis* sp. n. In addition, we provide COI barcode for identification in the future. The intraspecific genetic distance for *A. baotingensis* sp. n. based on the mean Kimura 2-parameter distance (K2P) and *p*-distance is 1.4% and 1.0%, respectively.

##### Atypus jianfengensis sp. n.

Taxon classificationAnimaliaAraneaeAtypidae

http://zoobank.org/82E29097-6474-4FC8-B3E7-22A31CE381FC

Figs 4 , 5

###### Type material.

**Holotype female (HN-2017-010)**: CHINA: Hainan Province: Ledong County, Jianfengling, Nantianchi, 18.74°N, 108.86°E, 823 m elevation, collected on 2 August 2017 by X. Xu, F. Liu, Z. Zhang, and D. Li (CBEE).

###### Paratypes.

4 females (HN-2017-003, HN-2017-005, HN-2017-007, HN-2017-008), collected at the same locality as the holotype, 2 August 2017 by X. Xu, F. Liu, Z. Zhang, and D. Li (CBEE). Male unknown.

###### Etymology.

The specific name refers to the type locality.

###### Diagnosis.

**Figure 4.**
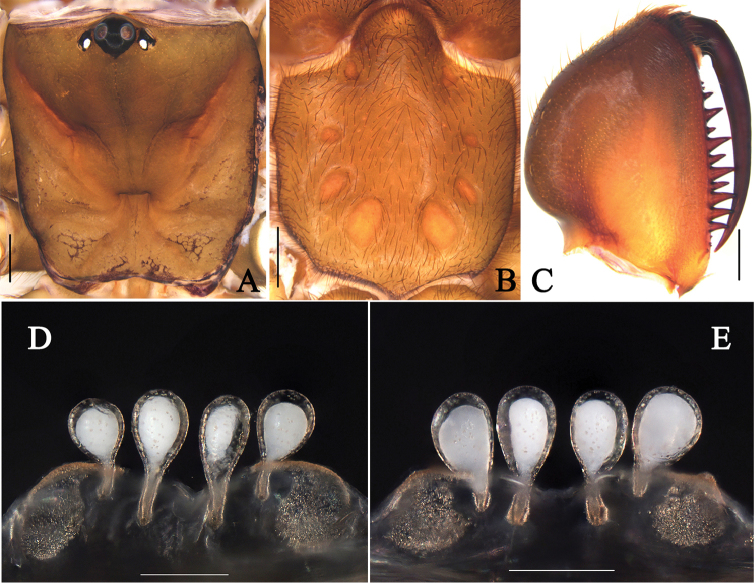
General somatic morphology and genital anatomy of *Atypus jianfengensis* sp. n. **A–D** female holotype (HN-2017-010) **E** female paratype (HN-2017-007) **A** carapace, dorsal view **B** labium and sternum, ventral view **C** left chelicera, inner-lateral view **D, E** vulva, dorsal view. Scale bars: 1 mm (**A–C**); 0.2 mm (**D–E**).

The female genitalia of the new species is similar to that of *A. karschi* Dönitz, 1887, but can be distinguished from the latter by the basal stalks of median pair of receptacula almost being as long as the diameter of their receptacula and much longer than the basal stalks of lateral pair (Fig. 4D–E); first pair of sigilla irregular shape (Fig. 4B).

###### Description.

**Female (holotype).** TL (including chelicerae) 18.74. CL 5.50, CW 4.97, AL 8.58, AW 5.98. Carapace red-brown. Eye region black. Eye diameters: AME 0.30, ALE 0.15, PME 0.16, PLE 0.14. Distances: AME–AME 0.27, AME–ALE 0.20, PME–PME 1.06, PME–PLE 0.12. MOA 0.29, front width 0.87, back width 1.38. Fovea transverse, occupying about 1/7 of carapace width at that point (Fig. 4A). Chelicerae orange red with 15 teeth on the promargin in a single row, two from apex small, basal three smallest (Fig. 4C). Labium wider than long, with a curved line marked in the middle. Sternum (Fig. 4B) light orange-brown, 4.80 long, 3.76 width, smooth, with scattered black hairs; sigilla deeply imprinted, first pair anteriorly pointed and irregular; fourth pair sub-oval, separated by nearly their width.

**Figure 5.**
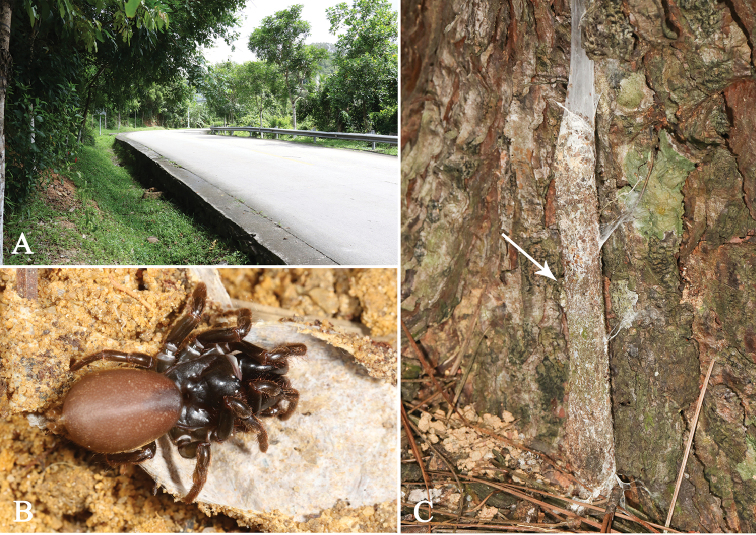
Microhabitat and general somatic morphology of *Atypus jianfengensis* sp. n. **A** microhabitat **B** female (HN-2017-010) **C** purse-web.

Abdomen, oval and medium brown (Fig. 5B), with a yellow dark dorsal tergite on anterior half. ALS 0.61, PMS 1.12, four-segmented PLS with lengths as follows: basal 0.77, median 0.69, subapical 0.62, apical 0.42, total 2.5.

Spines on all metatarsus; metatarsus IV with eleven dorsal spines. Leg formula: 1423.

Vulva (Fig. 4D–E): Genital atrium very short, pore patches large and rounded, the median pair of receptacula attached to the atrium more basally; the basal stalks of median pair of receptacula almost being as long as the diameter of their receptacula and much longer than the basal stalks of lateral pair.

**Table 3.** Leg measurements of *Atypus jianfengensis* sp. n., female.

	Femur	Patella	Tibia	Metatarsus	Tarsus	Total
I	3.71	1.94	2.07	2.08	1.55	11.35
II	3.24	1.55	1.25	1.40	1.33	8.77
III	2.71	1.72	0.67	0.64	0.60	6.34
IV	2.96	1.71	1.93	2.13	1.36	10.09

###### Variation.

Size range of females: carapace length 4.47–5.68, carapace width 4.02–5.15, total length 17.52–18.74, n = 5. Basal stalks of lateral pair of receptacula are much shorter in some specimens (Fig. 4E).

###### Habitat.

Purse webs were found attached to the base of pine trees.

###### Distribution.

Hainan Island (Jianfeng Mountain), China

###### GenBank accession numbers.

HN-2017-003: MH279550; HN-2017-005: MH279551; HN-2017-007: MH279552; HN-2017-008: MH279553; HN-2017-010: MH279554.

###### Remarks.

Although *A. jianfengensis* sp. n. is collected from Ledong County, Hainan Island, it can be diagnosed from *A. ledongensis* found at the same area, Jianfeng mountains, by the latter having very short basal stalks of median pair of receptacula. In addition, it can be distinguished from *A. baotingensis* sp. n. by the latter having upper incrassate basal stalks of the median pair of receptacula. The intraspecific genetic distance for *A. jianfengensis* sp. n. based on both the mean Kimura 2-parameter distance (K2P) and p-distance is 1.4% and 1.0%, respectively. The molecular data also provide the evidence that *A. jianfengensis* sp. n. can be distinguished from *A. baotingensis* sp. n. as well as from *A. ledongensis*: the interspecific genetic distance based on K2P and p-distance for the two new species are 15.4% and 13.8%, respectively, and between *A. jianfengensis* sp. n. and *A. ledongensis* are 17.2% and 15.3%, respectively.

## Supplementary Material

XML Treatment for Atypus baotingensis

XML Treatment for Atypus jianfengensis
